# Species-specificity of the secondary biosynthetic potential in *Bacillus*

**DOI:** 10.3389/fmicb.2023.1271418

**Published:** 2023-10-23

**Authors:** Qun-Jian Yin, Ti-Ti Ying, Zhen-Yi Zhou, Gang-Ao Hu, Cai-Ling Yang, Yi Hua, Hong Wang, Bin Wei

**Affiliations:** ^1^Fourth Institute of Oceanography, Ministry of Natural Resources, Beihai, China; ^2^College of Pharmaceutical Science and Collaborative Innovation Center of Yangtze River Delta Region Green Pharmaceuticals, Key Laboratory of Marine Fishery Resources Exploitation and Utilization of Zhejiang Province, Zhejiang University of Technology, Hangzhou, China

**Keywords:** *Bacillus*, genome mining, species-specificity, biosynthetic potential, natural products

## Abstract

**Introduction:**

Although *Bacillus* species have produced a wide variety of structurally diverse and biologically active natural products, the secondary biosynthetic potential of *Bacillus* species is widely underestimated due to the limited number of biosynthetic gene clusters (BGCs) in this genus. The significant variation in the diversity and novelty of BGCs across different species within the *Bacillus* genus presents a major obstacle to the efficient discovery of novel natural products from *Bacillus*.

**Methods:**

In this study, the number of each class of BGCs in all 6,378 high-quality *Bacillus* genomes was predicted using antiSMASH, the species-specificity of BGC distribution in *Bacillus* was investigated by Principal component analysis. Then the structural diversity and novelty of the predicted secondary metabolites in *Bacillus* species with specific BGC distributions were analyzed using molecular networking.

**Results:**

Our results revealed a certain degree of species-specificity in the distribution of BGCs in *Bacillus*, which was mainly contributed by siderophore, type III polyketide synthase (T3PKS), and transAT-PKS BGCs. *B. wiedmannii*, *B. thuringiensis*, and *B. cereus* are rich in RiPP-like and siderophore BGCs, but lack T3PKS BGCs, while *B. amyloliquefaciens* and *B. velezensis* are abundant in transAT-PKS BGCs. These *Bacillus* species collectively encode 77,541 BGCs, with NRPS and RiPPs being the two most dominant types, which are further categorized into 4,291 GCFs. Remarkably, approximately 54.5% of GCFs and 93.8% of the predicted metabolite scaffolds are found exclusively in a single *Bacillus* species. Notably, *B. cereus*, *B. thuringiensis*, and *B. velezensis* exhibit the highest potential for producing species-specific NRPS and PKS bioinformatic natural products. Taking two species-specific NRPS gene clusters as examples, the potential of *Bacillus* to synthesize novel species-specific natural products is illustrated.

**Conclusion:**

This study highlights the species-specificity of the secondary biosynthetic potential in *Bacillus* and provides valuable insights for the targeted discovery of novel natural products from this genus.

## 1. Introduction

*Bacillus* is a genus of Gram-positive, rod-shaped bacteria, a member of the phylum Firmicutes with 266 named species that are widely recovered from various environments such as soil, water, and air (Joshua and Rajasulocha, 2021). *Bacillus* can transform into oval endospores that are highly resistant to heat, cold, radiation, desiccation, and disinfectants. Consequently, *Bacillus* species have gained notoriety in the food industry as troublesome spoilage organisms, especially for some well-known pathogens (*Bacillus anthracis*, *Bacillus cereus*, and *Bacillus thuringiensis*) (Sansinenea and Ortiz, 2011; Christie and Setlow, 2020). *Bacillus* species have garnered growing interest due to their diverse applications in the fields of medicine, food, environment, and industry. One of the most notable types of natural products produced by the *Bacillus* species is lipopeptides, which have been widely used as antibiotics or natural bio-preservatives (Ongena and Jacques, 2008; Khurana et al., 2020). For instance, *Bacillus*-derived surfactins have garnered considerable attention as natural bio-preservatives due to their broad-spectrum antimicrobial activity, low toxicity, and environmentally friendly properties (Zhang et al., 2022). These studies suggest that the rational utilization of *Bacillus* resources holds significant importance in the prevention of pathogenic *Bacillus* species, as well as the development of novel bioresources.

According to the data from the Natural Products Atlas 2.0, *Bacillus* species have been reported to produce at least 455 secondary metabolites, primarily including lipopeptides, polyketides, bacteriocin, lantibiotic, siderophore, and macrolactone (van Santen et al., 2022; Xiao et al., 2022). These structurally diverse natural compounds exhibit a wide range of biological activities, including antimicrobial, antiviral, immunosuppressive, and antitumor activities (Sansinenea and Ortiz, 2011; Xiao et al., 2022). Due to the extensive research on natural products from *Bacillus* strains, the continued exploration of novel natural products from *Bacillus* poses significant uncertainties and challenges.

Genome mining approaches have been widely utilized for evaluating the biosynthetic potential of microorganisms and for the targeted discovery of novel natural products. The atlas of bacterial secondary metabolite biosynthetic gene clusters (BGCs) has been thoroughly investigated, unveiling the diversity and novelty of BGCs in all bacterial genera, including *Bacillus* (Wei et al., 2021; Gavriilidou et al., 2022). A recent large-scale genome mining analysis collectively identified 49,671 BGCs from 4,268 *Bacillus* genomes and revealed that the average number of BGCs per genome in *Bacillus* species reached 11.6, suggesting that a significant portion of the secondary metabolic potential of *Bacillus* species remains unexplored. However, there is significant variation in the number of BGCs among different strains within *Bacillus*, even in BGC-abundant species *Bacillus subtilis* (Xia et al., 2022). These studies indicate that in-depth analysis of the secondary metabolic potential of different species can contribute to more efficient research on the discovery of novel natural products from *Bacillus* strains. In addition, polyketide and peptidic natural products could be relatively accurately predicted from the sequences of the core biosynthetic enzymes in BGCs using bioinformatic tools, which has significantly promoted the discovery of bioactive synthesis-bioinformatic natural products (Blin et al., 2021; Wang et al., 2022).

Therefore, the present study first investigated the specificity of species-level distribution of BGCs in *Bacillus*. Then the structural diversity and novelty of the predicted secondary metabolites in *Bacillus* species with specific BGC distributions were analyzed to better understand the diversification of the secondary biosynthetic potential of *Bacillus* species. Finally, two representative gene cluster families (GCFs) was selected to exemplify the species-specificity of *Bacillus* strains in producing potential novel bioinformatic natural product.

## 2. Materials and methods

### 2.1. Genome dataset

A total of 7,793 available genomes from *Bacillus* species were downloaded from the NCBI database using the ncbi-genome-download script^1^ (February 2023). The GenBank accession number, genome size, and taxonomic information of all the genomes were obtained and summarized in Supplementary Table 1. Genome integrity was assessed by CheckM version 1.0.12 (Parks et al., 2015).

### 2.2. Biosynthetic gene cluster identification and networking

All high-quality genomes (completeness ≥95% and contamination ≤1%) were processed using the command-line version of antiSMASH 6.0 with the bacterial setting and otherwise default parameters (Blin et al., 2021). The number of each class of BGCs in each genome was extracted from the HTML files (antiSMASH outputs) using a custom Python kit^2^ with the following command “python3 ex_antismash_bgc.py <index.html_dir> output.xlsx”. All predicted BGCs were assigned to different biosynthetic GCFs using BiG-SLiCE with default settings (Kautsar et al., 2021) and summarized in Supplementary Table 2. The similarity network of the representative BGCs (the longest one) in each GCF was constructed using biosynthetic genes similarity clustering and prospecting engine (BiG-SCAPE) version 1.1.2 with the cut-off of 0.3 (Navarro-Muñoz et al., 2020) and visualized by Cytoscape version 3.8 (Shannon et al., 2003). The maximum likelihood phylogenetic tree based on genomes was constructed by the up-to-date bacterial core gene set (UBCG) pipeline and visualized in the interactive tree of life (iTOL) (Na et al., 2018; Letunic and Bork, 2021). The species-level phylogenetic tree was constructed based on the representative genome sequences using the same approach. The representative genome is the one with the highest assembly quality within that particular *Bacillus* species.

### 2.3. Molecular networking

All metabolite scaffolds predicted by antiSMASH were extracted from the JSON files (antiSMASH outputs) using a custom Python kit (see text footnote 2) with the following command “astool ex_smiles -i <json_dir> -o smiles.tsv -t antismash”. The source, BGC type, and smiles of each metabolite scaffold were summarized in Supplementary Table 3. The molecular similarity network of dereplicated metabolite scaffolds was generated by a custom python script^3^ based on the Tanimoto similarity. Metabolite scaffolds with a Tanimoto similarity score greater than 0.8 will be classified into the same cluster, and the final molecular similarity network was visualized using Cytoscape version 3.8.

## 3. Results and discussion

### 3.1. Species-specificity of BGC distribution in *Bacillus*

Among the 7,793 *Bacillus* genomes obtained from the NCBI reference genome database, 6,378 of them exhibited high assembly quality with completeness ≥95% and contamination ≤1%. Our recent research indicates that the assembly quality of bacterial genomes significantly affects the predicted number and completeness of BGCs within a genome using antiSMASH (Wei et al., 2023). Therefore, we only analyzed the distribution of BGCs in the 6,378 high-quality *Bacillus* genomes. These genomes collectively encode 77,541 BGCs, with an average of 12.2 BGCs per genome. The number of BGCs encoded in each genome varies greatly, ranging from 1 to 29 among different genomes (Supplementary Table 1).

**FIGURE 1:**
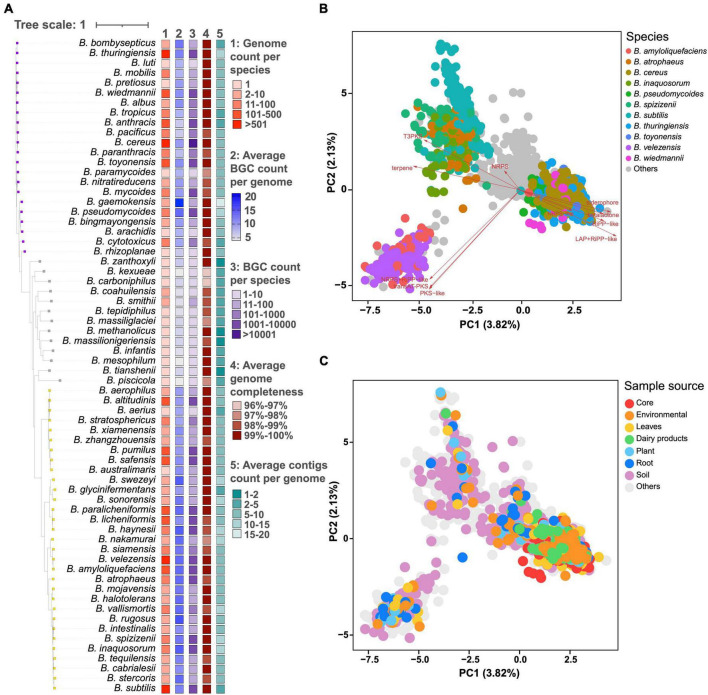
**(A)** The number of genomes, average, and total number of BGCs, average number of genome completeness and contigs per genome in 66 different *Bacillus* species. The phylogenetic tree was constructed based on the representative genome sequences for each species using the UBCG pipeline with the maximum likelihood method. These species are generally divided into three clades based on the average number of BGCs and taxonomic hierarchy, and are annotated with colored squares. Principal component analysis (PCA) of BGC distribution in 6,378 *Bacillus* genomes, selected genomes were highlighted in different colors according to their **(B)** species annotation and **(C)** sample source.

These 6,378 genomes belong to 66 distinct *Bacillus* species, among which 495 genomes have unknown species information. *Bacillus* species could be generally divided into three clades based on the average number of BGCs per genome and taxonomic hierarchy. Species in clades 1 and 3 have more genomic data and a larger average number of BGCs compared to that in clade 2. For example, all 21 *Bacillus* species with 10 or more genomes and having an average number of BGCs above 10 are from clades 1 and 3. *B. cereus* from clade 1 possesses 1,516 genomes and the average number of BGCs encoded in these genomes is 11.4 ± 3.2. *Bacillus velezensis* from clade 3 possesses 526 genomes and the average number of BGCs encoded in these genomes is 15.1 ± 3.4 (Figure 1A and Supplementary Table 4). Although there are only two genome data available for *Bacillus gaemokensis*, they encode 19 and 22 BGCs, respectively, which represents the most BGC-rich groups within the *Bacillus* genus. A recent influential study revealed that the *cereus* clade (*B. cereus, B. anthracis, B. thuringiensis*, etc.) possessed a moderate number of BGC as 11.7, which was highly consistent with our findings (Xia et al., 2022). Our findings offer a systematic comparison of the number of BGCs among *Bacillus* species, providing a valuable reference for the selection of promising *Bacillus* strains in natural product research. Additionally, it can be observed that the average completeness of genome in all *Bacillus* species reached 96%, with an average number of contigs less than 20. Moreover, the genome assembly quality of the bacterial species in clade 2 is not significantly lower than that in clades 1 and 3, indicating that the species-specificity of secondary metabolic potential in *Bacillus* species does exist and is independent of genome assembly quality.

Principal component analysis (PCA) demonstrated that the distribution of BGCs in *Bacillus* showed a certain degree of species-level specificity, which was mainly contributed by type III polyketide synthase (T3PKS), siderophore, and transAT-PKS BGCs (Figure 1B). In detail, *Bacillus amyloliquefaciens* and *B. velezensis* exhibited a very similar pattern in BGC distribution, displaying a pair of comparable PC1 and PC2 values. The genomes of *B. cereus*, *Bacillus pseudomycoides*, *B. thuringiensis*, *Bacillus toyonensis*, and *Bacillus wiedmannii* are scattered together in the PCA plot, thus having similar PC values. *Bacillus atrophaeus*, *Bacillus inaquosorum*, *Bacillus spizizenii*, and *B. subtilis* also showed very similar BGC composition and PC values (Supplementary Figure 1). In addition, BGC distribution in *Bacillus* showed a certain correlation with the source of the strains. For example, BGC distribution in *Bacillus* strains derived from core and dairy products exhibited some specificity, clustering in the lower right quadrant of the PCA plot (Figure 1C). On the other hand, *Bacillus* strains from soil exhibit the widest diversity in the composition of BGCs. However, PC1–PC5 only explained about 10% of the variations, suggesting that BGC distribution is pervasive in *Bacillus* (Supplementary Figure 2).

**FIGURE 2:**
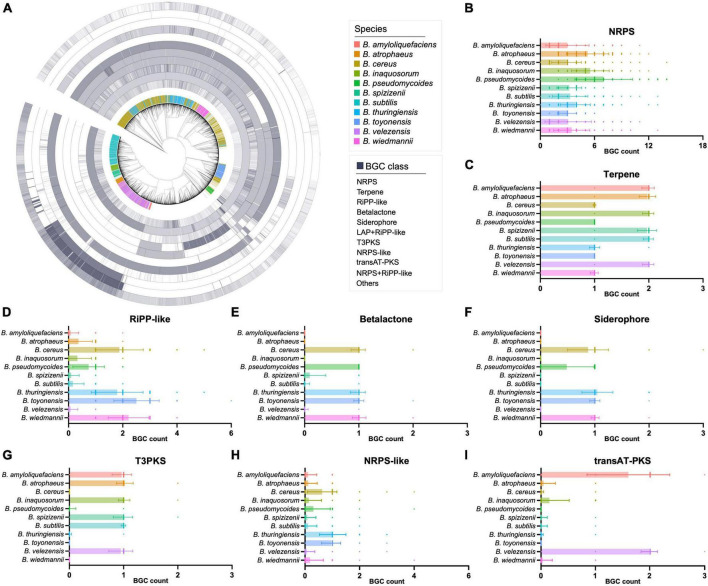
**(A)** The number of the major classes of BGCs in 6,378 *Bacillus* genomes. The 11 *Bacillus* species with specific BGC composition were highlighted in different colors in the first inner layer, others were presented in gray. The phylogenetic tree was constructed based on the genome sequences using the UBCG pipeline with the maximum likelihood method. The numbers of **(B)** NRPS, **(C)** terpene, **(D)** RiPP-like, **(E)** β-lactone, **(F)** siderophore, **(G)** T3PKS, **(H)** NRPS-like, and **(I)** trans-PKS BGCs in 11 selected *Bacillus* species.

The detailed composition of BGCs in these genomes was also presented in a circular heatmap (Figure 2A). It is worth noting that NRPS BGCs are widely distributed in these 11 selected *Bacillus* species with specific BGC distribution, with the average number of NRPS BGCs larger than 3 (Figure 2B). The result also suggested that some classes of BGCs showed significant variations between different *Bacillus* species. For instance, *B. amyloliquefaciens*, *B. atrophaeus*, *B. inaquosorum*, *B. spizizenii*, *B. subtilis*, and *B. velezensis* exhibit a high abundance of terpene and T3PKS BGCs, while they have fewer β-lactone, siderophore, and NRPS-like BGCs compared to the other five species (Figures 2C–I). In detail, terpene BGCs are also observed in all 11 *Bacillus* species, but the average number of these BGCs in six species is approximately twice that of the other species (Figure 2C). β-Lactone and siderophore BGCs are not detected in the genomes of *B. amyloliquefaciens* and *B. atrophaeus*, and T3PKS BGCs are not presented in *B. cereus*, *B. toyonensis*, and *B. wiedmannii*. These findings help understand the antagonism and phylogeny mechanisms of *Bacillus* and also contribute to the targeted exploration of specific types of natural products from *Bacillus*. Species-specificity of the secondary metabolic potential in actinobacterial genera *Streptomyces*, *Salinispora*, and *Amycolatopsis* have been reported to be associated with horizontal and vertical gene transfers (Jensen et al., 2007; Adamek et al., 2018; Xu et al., 2019). As shown in Figure 2A, BGC composition in some genomes of the selected *Bacillus* species showed significant variations from that of their counterparts, suggesting that both the horizontal and vertical gene transfers have influenced the evolution of secondary metabolic potential in *Bacillus* species.

### 3.2. Species-specificity of GCF distribution in *Bacillus*

**FIGURE 3:**
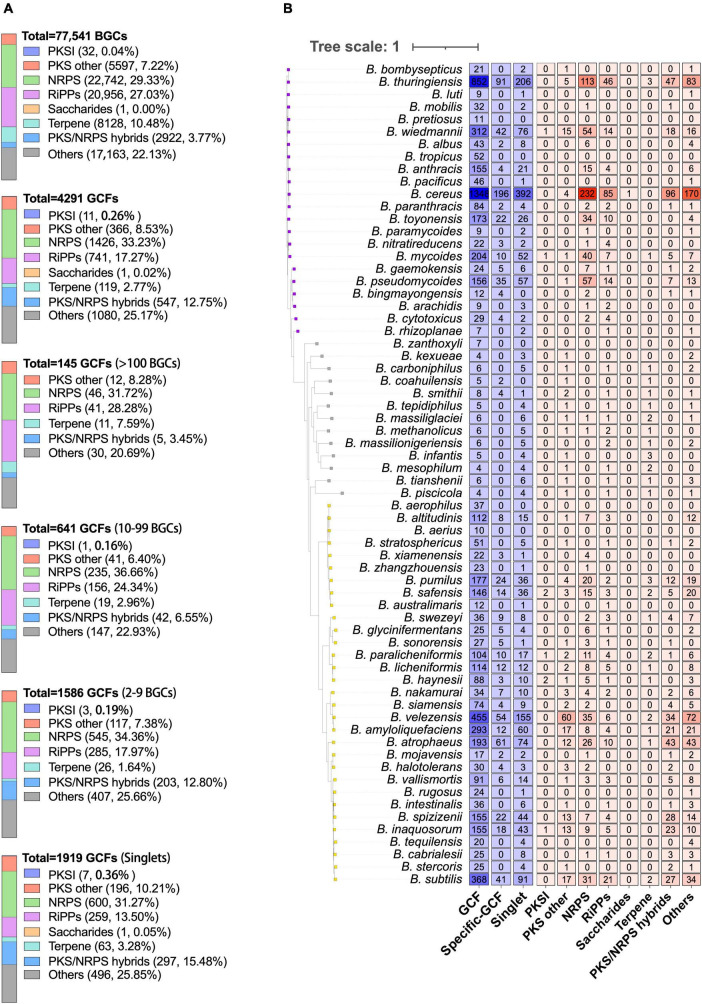
**(A)** The composition of different classes of BGCs or GCFs in *Bacillus* genomes. GCFs were obtained from all predicted BGCs using BiG-SLiCE with the default parameters (*T* = 300). GCFs were divided into four groups according to the number of BGCs in each GCF (1, 2–9, 10–99, and >100). **(B)** The numbers of GCFs, species-specific GCFs (≥2 BGCs), singlets, and different classes of species-specific GCFs (≥1 BGCs) in 66 different *Bacillus* species. Species-specific GCFs are defined as GCFs that are exclusively present in one *Bacillus* species. The phylogenetic tree was constructed based on the representative genome sequences for each species using the UBCG pipeline with the maximum likelihood method.

The 77,541 BGCs encoded by *Bacillus* species are categorized into 4,291 GCFs, which include 1,919 singlets, using the default threshold of 300 (Figure 3A). NRPS is the most prevalent class among both BGCs and GCFs, constituting 29.33% of the total BGCs and 33.23% of the total GCFs. The second most dominant class of GCFs in *Bacillus* species is RiPPs, organized into 741 GCFs, accounting for 17.27% of the total GCFs. Recently, Khurana et al. (2020) analyzed the distribution of BGCs in 178 *Bacillus* genomes and also found that NRPS and RiPPs are the two most predominant classes. It is worth noting that out of the extensive dataset of *Bacillus* gene clusters, only 32 PKSI and one saccharide BGCs were found, indicating a limited potential of *Bacillus* in synthesizing these natural products. Among the 4,291 GCFs, 1,919 of them consist of only one BGC, 1,586 GCFs possess 2–9 BGCs, 641 GCFs possess 10–99 BGCs (Figure 3A). Only 145 GCFs contain more than 100 BGCs and 24 GCFs contain more than 500 BGCs. Additionally, the composition of different classes of BGCs is very similar among these GCFs with different levels of popularity, for instance, NRPS and RiPPs continue to be the dominant categories, accounting for approximately 50% of the total GCFs (Figure 3A).

Among the obtained 4,291 GCFs, approximately 54.5% (2,337) of them are exclusively observed in one *Bacillus* species, including the 1,919 singlets. The number of species-specific GCFs in 66 different *Bacillus* species was presented in Figure 3B and Supplementary Table 4, revealing that *B. cereus* encoded the largest number of species-specific GCFs (588, 43.62%), followed by *B. thuringiensis* (297, 34.86%), and *B. velezensis* (209, 45.93%). The ratio of species-specific GCFs in these species did not exhibit a proportional correlation with the number of genomes in each species, suggesting that *B. velezensis* possessed the highest abundance of species-specific biosynthetic genes. The number of genomes in *Bacillus* species is significantly positively (*R*^2^ = 0.8932, *p* < 0.0001) correlated with the number of species-specific GCFs (Supplementary Figure 3), which partially explains the highest number of species-specific GCFs in these three species. It is worth noting that among the 66 species of *Bacillus*, all 11 species with specific BGC distribution have several species-specific GCFs that rank within the top 15. The species-specific GCFs in *B. cereus* and *B. thuringiensis* mainly belong to NRPS, RiPPs, and PKS-NRPS hybrid types, while those in *B. velezensis* mainly belong to PKSother, NRPS, and PKS-NRPS hybrid types (Figure 3B). These findings provide valuable information for the targeted discovery of unique RiPPs and PKSother natural products from *Bacillus* species. These species-specific gene clusters may be associated with their adaptation to the respective environmental conditions. The diversity and specificity of BGC distribution in six clades of *Bacillus* species were also investigated and revealed the dominant and clade-specific BGCs in the representative *Bacillus* genomes (Xia et al., 2022). This present study analyzed the diversity and specificity of BGCs in *Bacillus* at the species level, which provides higher precision. However, for some species with fewer genomic sequences, the results need further validation.

**FIGURE 4:**
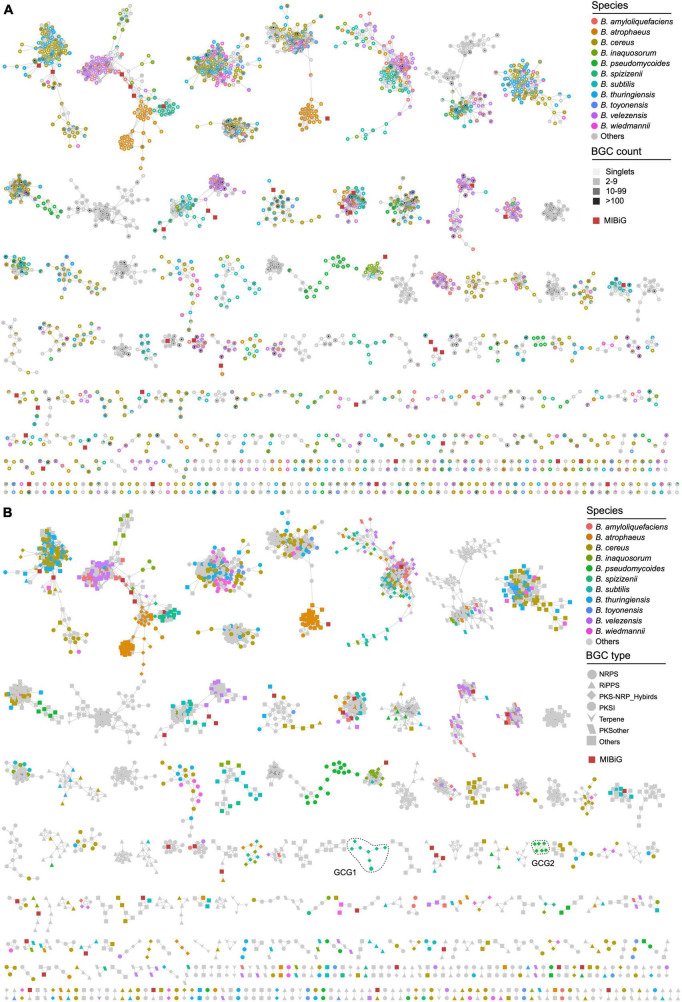
Gene cluster network of the representative BGCs of 4,291 GCFs (1,513 singlets not shown). The network was generated using BiG-SCAPE with the default parameters (threshold = 0.3). Species-specific BGCs are colored according to their species-level classification, other BGCs are presented in gray. **(A)** The fill color of the nodes reflects the BGC count in each GCF. **(B)** The shape of the nodes reflects the class of BGCs. BGCs sourced from the MIBiG database are depicted as dark red squares.

Due to the incompatibility of BIG-SCAPE for constructing a network of a large number of gene clusters, the longest gene cluster in each GCF was selected as the representative BGC for building the gene cluster network of *Bacillus* species. The remaining 4,291 BGCs have a similarity higher than the threshold of 0.3 (BIG-SCAPE) with 42 characterized BGCs from the MIBiG database, thereby collectively forming 417 gene cluster groups (GCGs), including 1,513 singlets (Figure 4A and Supplementary Table 2). Thirty-six out of the 417 GCGs contained 42 known BGCs, including BGCs responsible for the biosynthesis of bacillomycin D, bacilysin, and bacillibactin, et al., suggesting that the 258 homologous BGCs distributed within GCGs containing BGCs from the MIBiG database are highly likely to possess the potential for synthesizing analogs of these reported antibiotics. The remaining 381 (91.4%) GCGs exhibit low similarity (less than 0.3) to characterized BGCs, thus showing a high potential for synthesizing novel secondary metabolites. The majority of the GCGs contained representative BGCs from multiple *Bacillus* species, indicating that the specificity of these BGCs is not strong enough. These BGCs are widely distributed in the *Bacillus* genus, and their encoded natural products may have similar functions. It is worth noting that all representative BGCs in some GCGs are solely derived from one *Bacillus* species, such as *B. pseudomycoides* and *B. spizizenii* (Figure 4A). These BGCs with a higher degree of species-specificity hold significant value in the fields of natural product discovery and research on the evolutionary mechanisms of *Bacillus*.

Representative BGCs of each GCF in the gene cluster network were also presented according to their categories, which revealed the composition of different types of GCFs in *Bacillus* species. The findings suggest that the representative BGCs primarily belong to the NRPS and other categories, with a significant presence in *B. cereus*, *B. thuringiensis*, and *B. atrophaeus* (Figures 3, 4B). All GCGs with more than eight representative BGCs are not species-specific and contain BGCs from two or more *Bacillus* species. The largest species-specific GCG primarily comprises PKS-NRPS hybrid BGCs (GCG1) derived from *B. spizizenii*, while the second largest species-specific GCG also comprises PKS-NRPS hybrid BGCs (GCG2), but derived from *B. pseudomycoides*. Other species-specific GCGs contained 2–3 representative BGCs and were mainly derived from *B. cereus*, *B. thuringiensis*, and *B. subtilis*. In addition, some BGCs are clustered together at the species level, such as some PKS-NRPS hybrid BGCs from *B. atrophaeus* (Figure 4B), and their in-depth exploration holds significant research value. Furthermore, it is noteworthy that all 1,513 singlets are species-specific BGCs, predominantly falling into the NRPS and RiPPs categories. These BGCs are mainly derived from *B. pseudomycoides* and *B. cereus* (Supplementary Table 2). These singlets (orphan gene clusters) represent a tremendous source of novel and bioactive natural products.

### 3.3. Species-specificity of predicted metabolite scaffolds in *Bacillus*

**FIGURE 5:**
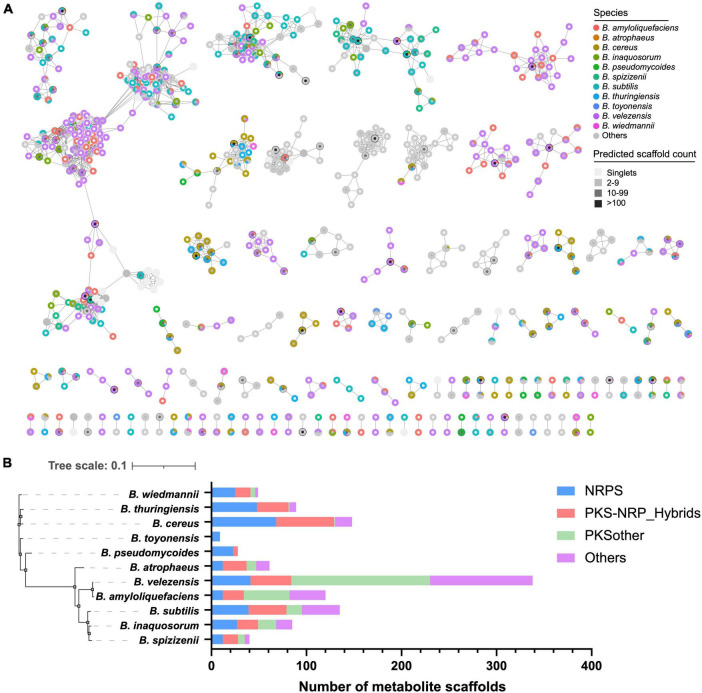
**(A)** Molecular network of the 1,442 unique predicted metabolite scaffolds obtained from *Bacillus* genomes. Species-specific metabolite scaffolds are colored according to their species-level classification, metabolite scaffolds from other species or multiple species are presented in gray. The fill color of the nodes reflects the frequency of the metabolite scaffold predicted from *Bacillus* genomes. **(B)** The composition of different classes of species-specific metabolite scaffolds in 11 selected *Bacillus* species.

To further explore the species-specificity of the secondary biosynthetic potential of *Bacillus*, we compared the diversity and specificity of the predicted metabolite scaffolds encoded mainly by NRPS and PKS gene clusters in *Bacillus*. A total of 33,996 metabolite scaffolds were obtained from the JSON files (antiSMASH outputs), due to the high homology of many BGCs, a large part of the metabolite scaffolds predicted based on these BGCs are identical. Among them, five metabolite scaffolds have been obtained over 1,000 times, and 47 metabolite scaffolds have been obtained over 100 times (Figure 5A and Supplementary Table 3). A total of 1,442 unique metabolite scaffolds were retained after dereplication, which was organized into 109 molecular families, including 716 singlets, at a Tanimoto similarity score of 0.8. Some predicted products contain uncertain structural units, denoted by asterisks, which may affect the analysis of the structural diversity of these products. It is worth noting that some metabolite scaffolds are clustered together at the species level, such as certain scaffolds found in *B. velezensis* (Figure 5A). These species-specific natural products are worth obtaining through targeted approaches such as synthetic-bioinformatic natural products (syn-BNP) or heterologous expression of gene clusters (Scherlach and Hertweck, 2021). The relationship between their specificity and physiological activities is also worth exploring. As shown in Figure 5B, the predicted metabolite scaffolds are mainly encoded by NRPS and PKS BGCs. Among the 1,442 unique metabolite scaffolds, 1,352 of them are exclusively observed in one *Bacillus* species. *B. velezensis* was found to possess the largest number of species-specific metabolite scaffolds (338), followed by *B. cereus* (148) and *B. subtilis* (135). The composition of different categories of species-specific metabolite scaffolds in 11 selected *Bacillus* species demonstrated that *B. velezensis* encoded more species-specific PKSother natural products while *B. cereus* and *B. subtilis* have the potential to synthesize more species-specific NRPS and PKS/NRPS hybrid natural products. The inter- and intraspecies distribution of BGCs in *B. subtilis* have been systematically investigated by Steinke et al., and revealed that multiple GCFs are species or clade specific and the variations in the structures of some BGCs are conserved within phylogenetically related isolates (Steinke et al., 2021). The finding may partially explain the presence of a higher number of species-specific GCFs and bioinformatic natural products in *B. subtilis*.

**FIGURE 6:**
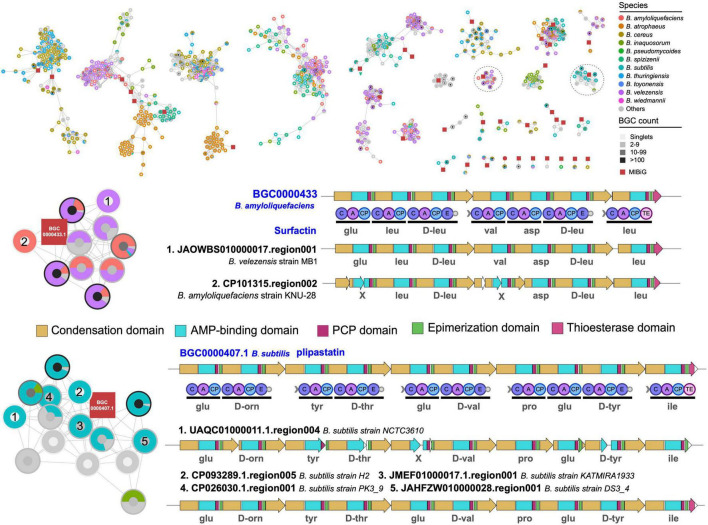
Gene cluster network of the representative BGCs. Only gene cluster groups containing known BGCs from the MIBiG database were retained. Comparative analysis of two gene cluster group containing known BGCs and BGCs encoding species-specific metabolite scaffolds. The predicted substrates for each adenylation domain are displayed underneath.

More detailed analysis of the structures of BGCs in two selected GCGs revealed that *B. velezensis* showed high potential to produce surfactin-like natural products, and *B. subtilis* showed capacity to produce plipastatin-like natural products (Figure 6). In addition, the BGCs were divided into different GCFs in BIG-SLICE analysis and were exclusively observed in *B. velezensis* and *B. subtilis*, respectively, suggesting the presence of significant variations in biosynthetic genes within these BGCs, which may have an impact on the structure of the final natural products. Surfactin and plipastatin have been reported to exhibit a broad range of potent antibacterial activities (Abdel-Mawgoud et al., 2008; Zhou et al., 2023). Using the culture-independent syn-BNP strategy, it is expected to rapidly obtain novel surfactin-like or plipastatin-like pseudo-natural products (Wang et al., 2022), which represents one of the most important research directions for the efficient discovery of natural products.

## 4. Conclusion

The present study first compared the numbers of different classes of BGCs in 6,378 high-quality *Bacillus* genomes of 66 different species, revealing that *Bacillus* exhibit a certain degree of species-specificity in BGC distribution, and the specificity was mainly contributed by siderophore, T3PKS, and transAT-PKS types of BGCs. The species-specificity of secondary biosynthetic potential in *Bacillus* was further explored by comparing the distribution of different classes of GCFs and predicted metabolites scaffolds. The findings revealed that about 54.5% of GCFs and 93.8% of predicted metabolite scaffolds are observed in only one *Bacillus* species. These GCFs were primarily associated with NRPS and PKS natural products. *B. cereus* and *B. thuringiensis* exhibited the highest potential for producing species-specific NRPS natural products, whereas *B. velezensis* demonstrated the highest potential for synthesizing species-specific PKS natural products. Taking two species-specific NRPS BGCs as examples, the potential of *Bacillus* to synthesize novel species-specific natural products is illustrated. The present study systematically reveals the species-specificity of the secondary biosynthetic potential in *Bacillus* and provides valuable insights for the targeted discovery of novel natural products from *Bacillus*.

## Data availability statement

The datasets presented in this study can be found in online repositories. The names of the repository/repositories and accession number(s) can be found in the article/Supplementary material.

## Author contributions

Q-JY: Funding acquisition, Investigation, Writing – original draft. T-TY: Investigation, Visualization, Writing – original draft. Z-YZ: Data curation, Methodology, Software, Writing – review and editing. G-AH: Methodology, Software, Writing – review and editing. C-LY: Data curation, Software, Writing – review and editing. YH: Data curation, Software. HW: Funding acquisition, Supervision, Writing – review and editing. BW: Conceptualization, Funding acquisition, Supervision, Writing – review and editing.

## Footnotes

1
https://github.com/kblin/ncbi-genome-download

2
https://github.com/BioGavin/astool/

3
https://github.com/BioGavin/wlabkit/blob/master/astool/script/network/CSS.py
