# Supplementary material for: Species-specificity of the secondary biosynthetic potential in Bacillus
Source: Front Microbiol. 2023 Oct 23;14:1271418. doi: 10.3389/fmicb.2023.1271418 (PMC10626522; doi:10.3389/fmicb.2023.1271418)
Supplement: Supplementary file 1 [file Data_Sheet_1.DOCX]

Supplementary Material

# Supplementary Tables

**Table S1**. The genomic features of 6378 high-quality *Bacillus* genomes and the numbers of various classes of BGCs divided by antiSMASH and BIG-SCAPE, respectively.

**Table S2**. Information about the representative BGCs of each GCF and the resulting gene cluster groups.

**Table S3**. Information about the predicted metabolite scaffolds encoded by *Bacillus* BGCs and the number of species-specific scaffolds in 11 selected *Bacillus* species.

**Table S4**. The number of genomes, average and total number of BGCs, average number of genome completeness and contigs per genome in 66 different *Bacillus* species.

# Supplementary Figures


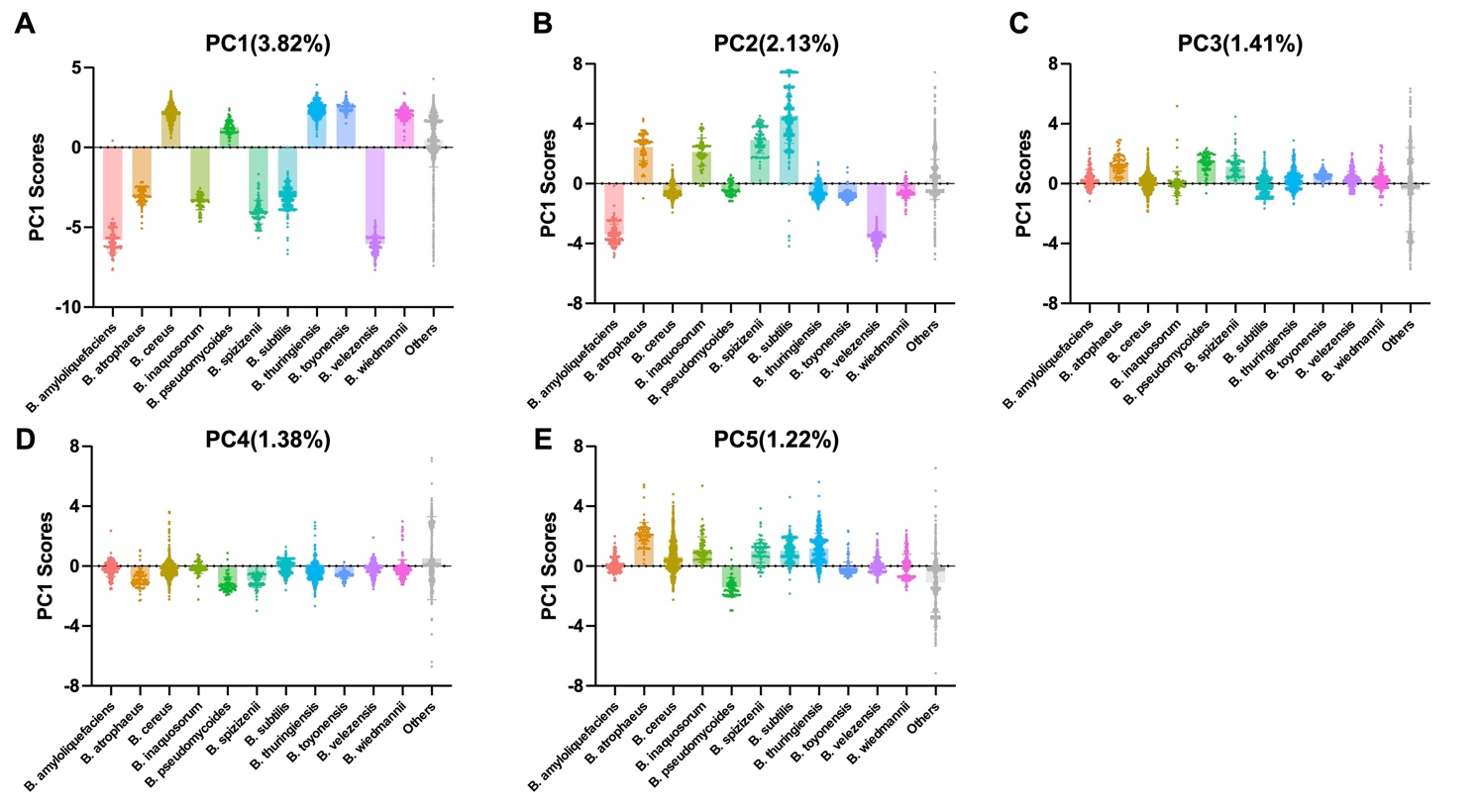


**Supplementary Figure 1.** The Principal Component values of genomes in 11 *Bacillus* species with specific BGC distribution. (**A**) PC1, (**B**) PC2, (**C**) PC3, (**D**) PC4, and (**E**) PC5.


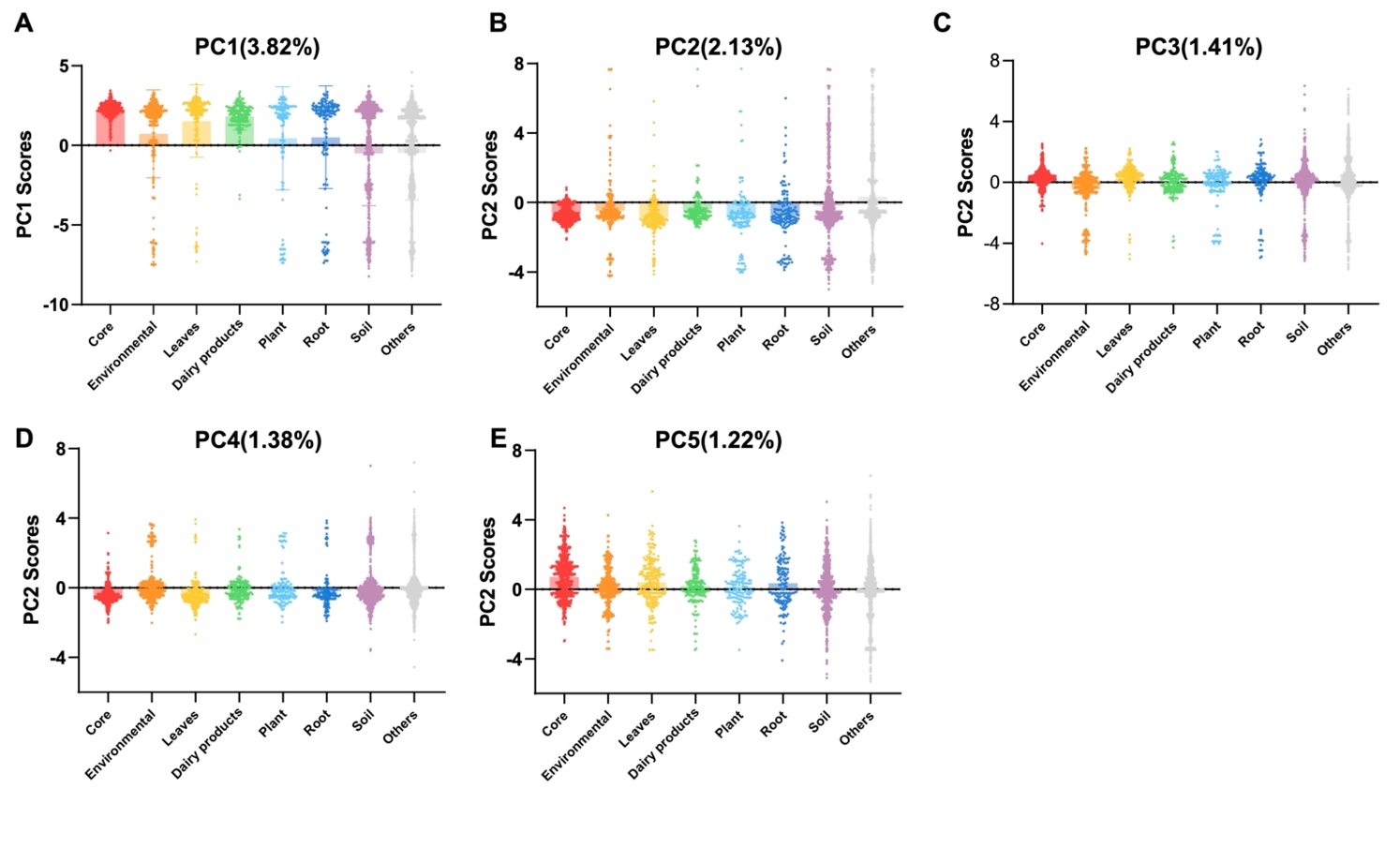


**Supplementary Figure 2.** The Principal Component values of genomes in *Bacillus* strains derived from different source. (**A**) PC1, (**B**) PC2, (**C**) PC3, (**D**) PC4, and (**E**) PC5.

**
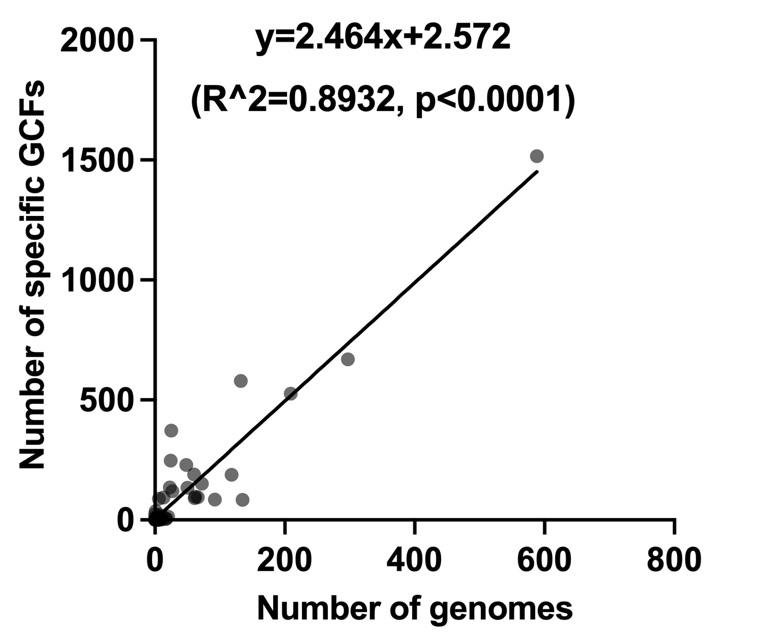
**

**Supplementary Figure 3.** Leaner correlation analysis of the number of genomes in each *Bacillus* species with the number of species-specific GCFs within each species.
